# Cloned HTLV-1+CD4+, but not CD8+, T-cells display an oncogenic miRNome

**DOI:** 10.1186/1742-4690-8-S1-A169

**Published:** 2011-06-06

**Authors:** Céline Vernin, Christiane Pinatel, Nicolas Nazaret, Eric Wattel, Catherine Legras-Lachuer, Franck Mortreux

**Affiliations:** 1 Oncovirologie et de Biotherapies, UMR5239 CNRS/ENS Lyon/UCBL/HCL, Hopital Pierre Benite, Lyon, France; 2 Oncovirologie et de Biotherapies, Centre Léon Berard, Lyon, France; 3 ProfileXpert, Neurobiotec Service, Bron, 69500, France

## 

HTLV-1 persistence in vivo relies on the persistent clonal expansion of its host cells. These are CD4+ and CD8+ T cells, yet ATL is regularly CD4+. Accordingly, untransformed HTLV-1+CD4+ but not CD8+ T cells cloned from carriers cumulate the features of preleukemic cells, including multinuclearity, chromatin bridges, increased cell cycling and inappropriate telomerase activity. MicroRNAs (miR) modify the maturation of a plethora of T-cells RNA and their deregulation would therefore constitute an appropriate explanation for the Tax-dependent or -independent pleiotropic changes in the phenotype of HTLV-1+CD4+ T cells. As the miRNome of naturally infected untransformed cells has not been investigated to date, we assessed the miR expression profiling of T cells cloned from carriers. Microarray results, confirmed by quantitative RTPCR, showed that, upon infection, CD4+ and CD8+ clones yielded aberrant expression of 15 distinct miRs including miR-34b and miR-494 that were respectively over- and underexpressed in both compartments. The more prominent effect of the infection consisted in the CD4+-restricted overexpression of the cancer-related miRs miR-21, -27b and -23b associated with the CD4+-restricted downregulation of the proapoptotic miR-15 and -16. Data were extended by the analysis of 40 additional CD4+ clones (20 infected). Crossing the miRNome against the whole transcriptome data identified putative miR-targeted genes. In silico, those targeted by miR-23b and -27b defined 2 hitherto unknown pathways involving the cell cycle and genetic disorders. Therefore HTLV-1 triggers a phenotype-specific miR signature consistent with the preleukemic HTLV-1+CD4+ phenotype.

